# Potential Roles of Intrinsic Disorder in Maternal-Effect Proteins Involved in the Maintenance of DNA Methylation

**DOI:** 10.3390/ijms18091898

**Published:** 2017-09-04

**Authors:** Hongliang Liu, Qing Wei, Chenyang Huang, Yong Zhang, Zekun Guo

**Affiliations:** 1 College of Veterinary Medicine, Northwest A&F University, Yangling 712100, China; liuhl355113@sina.com (H.L.); xwq3519@sina.com (Q.W.); cherry9332@163.com (C.H.); zhangyong1956@nwsuaf.edu.cn (Y.Z.); 2 Key Laboratory of Animal Biotechnology, Ministry of Agriculture, Northwest A&F University, Yangling 712100, China; 3 College of Eco-Environmental Engineering, Qinghai University, Xining 810016, China

**Keywords:** PGC7/DPPA3/STELLA, ZFP57, TRIM28/KAP1/TIF1β, DNMT1, intrinsic disorder, protein–protein interaction

## Abstract

DNA methylation is an important epigenetic modification that needs to be carefully controlled as a prerequisite for normal early embryogenesis. Compelling evidence now suggests that four maternal-effect proteins, primordial germ cell 7 (PGC7), zinc finger protein 57 (ZFP57), tripartite motif-containing 28 (TRIM28) and DNA methyltransferase (cytosine-5) 1 (DNMT1) are involved in the maintenance of DNA methylation. However, it is still not fully understood how these maternal-effect proteins maintain the DNA methylation imprint. We noticed that a feature common to these proteins is the presence of significant levels of intrinsic disorder so in this study we started from an intrinsic disorder perspective to try to understand these maternal-effect proteins. To do this, we firstly analysed the intrinsic disorder predispositions of PGC7, ZFP57, TRIM28 and DNMT1 by using a set of currently available computational tools and secondly conducted an intensive literature search to collect information on their interacting partners and structural characterization. Finally, we discuss the potential effect of intrinsic disorder on the function of these proteins in maintaining DNA methylation.

## 1. Introduction

DNA methylation is among the best studied epigenetic modifications and plays critical roles in a broad range of biological processes [1,2,3]. During gametogenesis and early embryogenesis, DNA methylation undergoes a process of erasure, acquisition and maintenance. Disruptions in any of these steps may lead to dysregulation of DNA methylation, resulting in severe early embryogenesis defects. Recent studies have shown that four maternal-effect proteins are important for the maintenance of DNA methylation during early embryogenesis. In the zygote, PGC7 functions as a protector against active demethylation of the maternal genome and imprinted genes [4,5]. During cleavage divisions, ZFP57, TRIM28 and DNMT1 provide protection for imprinted genes from passive demethylation [6,7,8].

Intrinsically disordered proteins (IDPs) are functional proteins that do not fold into stable secondary and tertiary structures under physiological conditions, either in their entirety or in part [9,10,11,12,13,14]. The disordered proteins or intrinsically disordered protein regions (IDPRs) have exposed short linear peptide motifs and interaction-prone structural motifs (Molecular Recognition Features, MoRFs), which allow IDPs to have the ability to adapt to a variety of binding partners to perform their function. In fact, many studies have shown that IDPs play critical roles in the regulation of intracellular signalling and crucial cellular processes, including regulation of transcription and cell-cycle control [10,11,12,15,16,17,18,19,20,21]. Moreover, the IDPs or IDPRs are often extensively decorated with diverse post-translational modifications, which facilitate regulation of their function and stability in cells [22,23,24,25,26]. In this study, we show that four maternal-effect proteins (PGC7, ZFP57, TRIM28 and DNMT1) that are associated with DNA methylation maintenance have different levels of intrinsic disorder and that this disorder plays an important role in regulating their function. Additionally, knowledge of the protein–protein interaction network generated by the four maternal-effect proteins and their partners will likely provide a substantial resource for subsequent in-depth studies of their functions.

## 2. Results and Discussion

### 2.1. Primordial Germ Cell 7 (PGC7)

PGC7 (also known as DPPA3 or STELLA) is a maternal-effect factor that plays a critical role in normal early development [27,28,29]. Since Nakamura et al. (2007) [4] found that PGC7 protects the DNA methylation state of several imprinted genes during epigenetic reprogramming after fertilisation, a series of related studies have demonstrated that PGC7 is involved in the regulation of DNA methylation [5,30,31,32,33]. Here, we evaluate the intrinsic disorder propensities of PGC7 using a broad set of computational tools and discuss the effect of intrinsic disorder on PGC7 for maintenance of DNA methylation.

**Figure 1:**
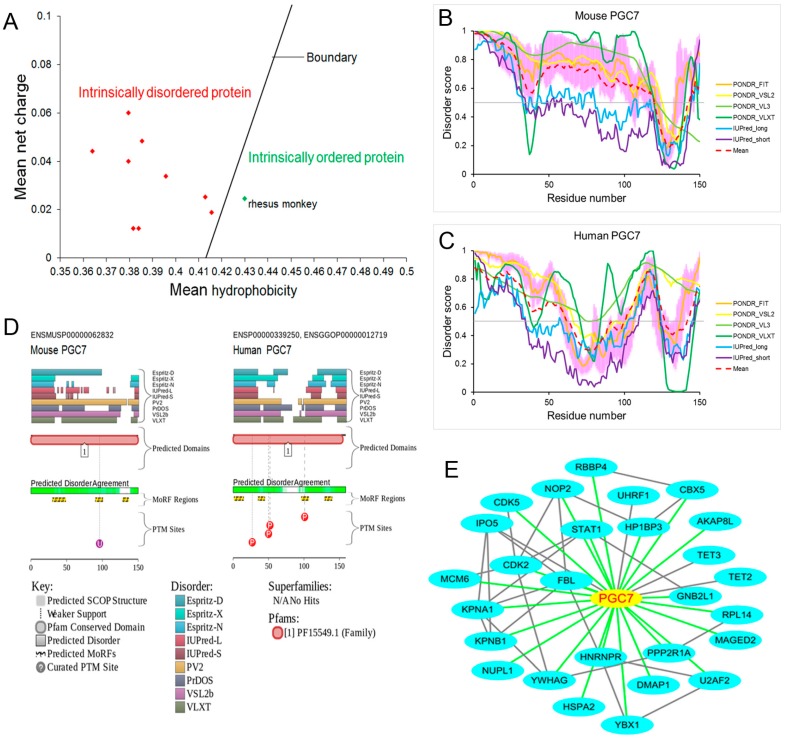
Evaluating functional intrinsic disorder and interactability of primordial germ cell 7 (PGC7). (**A**) Computational analysis (charge–hydrophobicity (CH) plot) of 10 mammalian PGC7 proteins. Intrinsically disordered and ordered proteins are separated by a linear boundary, which is described by the following relationship: Hb (mean hydrophobicity) = (R (mean net charge) + 1.151)/2.785. The mean hydrophobicity and mean net charge of PGC7 proteins were calculated with PONDR (http://www.pondr.com/). The green dot indicates the position of rhesus monkey PGC7 protein in the CH plot, the red dot indicates the position of another nine mammalian PGC7 proteins. (**B**,**C**) Evaluating intrinsic disorder propensity of mouse PGC7 (UniProt ID: Q8QZY3) and human PGC7 (UniProt ID: Q6W0C5) by series of per-residue disorder predictors. Disorder profiles generated by PONDR^®^FIT, PONDR^®^VSL2, PONDR^®^VL3, PONDR^®^VLXT, IUPred-long, and IUPred-short are shown by *orange*, *yellow*, *light green*, *green*, *light blue* and *purple* lines, respectively. *Dark red dashed line* shows the mean disorder propensity calculated by averaging disorder profiles of above six predictors. *Light pink* shadow around the PONDR^®^FIT shows error distribution. The predicted disorder scores above the 0.5 threshold (the grey line) are considered as the disordered residues/regions. Detailed statistical information is listed in supplemental Table S1. (**D**) Intrinsic disorder propensity and disorder-related functional information generated for mouse and human PGC7 by the D^2^P^2^ database. Here, the *green-and-white bar* in the middle of the plot shows the predicted disorder agreement between nine predictors. The *green* parts are disordered regions (75% of predictor ’s agree). *Yellow bar* shows the location of the predicted disorder-based binding sites (molecular recognition features, MoRFs). *Coloured circles* at the bottom of the plot show the location of various post-translational modifications (PTMs). (**E**) The interaction network of PGC7 with its partners. The network contains 28 nodes and 51 edges. Here, four proteins (IPO5, UHRF1, TET2 and TET3) are previously reported, 23 proteins are newly validated by our research team. The STRING online tool was used to determine the known interactions between PGC7 and its partners. We further visualized the network in Cytoscape and grafted our identified interactions onto it. The *grey edges* show the interaction obtained from the STRING database, whereas *green edges* show the interaction discovered by our research term.

PGC7 is a small protein, comprising 150 amino acids in mice and 159 amino acids in humans. Interestingly, the PGC7 protein only exists in mammals and evolved very quickly [28,34,35]. Thus, we first chose to analyse the amino acid composition of PGC7 from 10 representative species. As shown in Table 1, although the amino acid sequence is poorly conserved across mammalian species, all 10 mammalian PGC7 proteins contain a higher proportion of disorder-promoting residues (51.33–58.06%) and a low content of order-promoting residues (27.70–33.33%). In the absence of experimental assignments, a CH (charge–hydrophobicity) plot method (described by Uversky et al. (2000) [16]) has been developed for the separation of IDPs and intrinsically ordered proteins. The IDPs are separated from ordered proteins by a linear boundary, with disordered proteins above the boundary and ordered proteins below. This boundary is described by the relation: Hb (mean hydrophobicity) = (R (mean net charge) + 1.151)/2.785. As shown in the CH plot (Figure 1A), nine mammalian PGC7 proteins are located in the region of intrinsically disordered proteins, only rhesus monkey PGC7 protein is below the boundary.

PGC7 has been best described in mice and humans as an important maternal factor that participates in protecting DNA methylation. Thus, we next chose to evaluate the intrinsic disorder propensities of mouse and human PGC7 proteins using four algorithms from the predictor of natural disordered regions (PONDR) family [36,37,38,39,40], as well as the IUPred web server [41]. According to the PONDR^®^FIT, PONDR^®^VSL2, PONDR^®^VL3, PONDR^®^VLXT, IUPred-long and IUPred-short analyses, mouse PGC7 is characterised by contents of the predicted disordered residues (CPDR) of 90.67%, 87.33%, 80.67%, 78.67%, 50.67% and 32.67%, respectively (Figure 1B). Human PGC7 is characterised by CPDRs of 62.26%, 79.25%, 100.00%, 74.84%, 45.28% and 38.36%, respectively (Figure 1C). Overall, 84.67% of mouse PGC7 and 67.30% of human PGC7 residues are predicted to be disordered (disorder scores > 0.5), based on averaging the outputs of the above predictors. Figure 1B also shows that the mouse PGC7 protein is highly disordered. Only the C-terminal tail (residues 120–142) is predicted to be ordered. Figure 1C shows that human PGC7 is predicted to have several IDPRs (residues 1–61, 100–126 and 143–159).

We further analysed the disorder status of PGC7 proteins using the MobiDB database (http://mobidb.bio.unipd.it/) [42,43], which provides consensus disorder scores by aggregating the output from 10 predictors. The consensus MobiDB CPDR values of mouse PGC7 (UniProt ID: Q8QZY3) and human PGC7 (UniProt ID: Q6W0C5) are 46.00% and 56.86%, respectively. If the classification of proteins based on their CPDR values is used, where proteins are considered as highly ordered (CPDR < 10%), moderately disordered (10% ≤ CPDR < 30%), or highly disordered (CPDR ≥ 30%) [44], mouse and human PGC7 would be placed into the category of highly disordered proteins.

To further illustrate the disorder predispositions of mouse and human PGC7 proteins, Figure 1D shows the output of the D2P2 database (http://d2p2.pro/) [45]. The percent of disordered regions (75% of predictors agree) in mouse and human PGC7 proteins are 40.67% and 46.54%, respectively. Further, mouse PGC7 is expected to have three disorder-based binding sites (residues 30–48, 90–100 and 128–136) and a ubiquitination site. In human PGC7 protein, there are four disorder-based binding sites (residues 1–14, 35–44, 96–106 and 129–139) and four phosphorylation sites. Unfortunately, there is no published literature that has examined the effect of post-translational modification on the function of PGC7.

According to the output of the D^2^P^2^ database, we also found that 24% of the mouse PGC7 and 27% of the human PGC7 protein length are covered by disorder-based binding regions, predicting that PGC7 utilises a disorder-based binding mode for interaction with its partners. In fact, we identified 290 previously unknown PGC7-interacting proteins from human embryonic kidney (HEK-293T) cells using a streptavidin–biotin affinity purification technique, combined with LC−MS/MS. Figure 1E represents the interaction network of PGC7 with its partners. In this network, only four proteins, including importin 5 (IPO5), ubiquitin like with PHD and ring finger domains 1 (UHRF1), tet methylcytosine dioxygenase 2 (TET2) and tet methylcytosine dioxygenase 3 (TET3), were previously reported. The remaining 23 proteins were newly validated by our research team [46]. 

**Figure 2:**
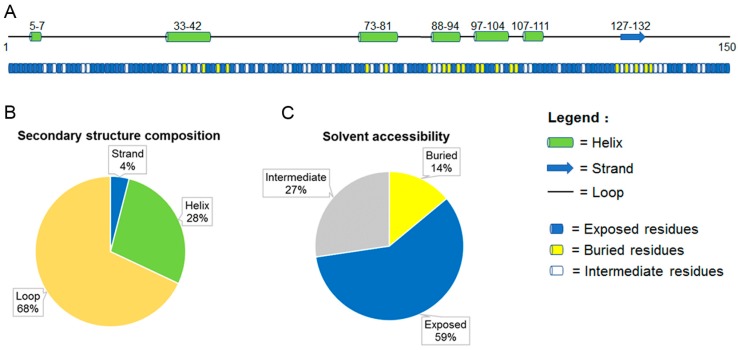
Secondary Structure and Solvent Accessibility Prediction for mouse primordial germ cell 7 (PGC7) (UniProt ID: Q8QZY3). (**A**) The secondary structure and solvent accessibility information generated for mouse PGC7 by PredictProtein server (https://www.predictprotein.org/). The predicted secondary structures are shown in upper part of the figure. Three states of secondary structure are predicted: helix (includes α-, π - and 3_10-helix), strand (extended strand in β-sheet conformation of at least two residues length) and loop. The prediction of solvent accessibility for all residues are shown in the lower part of the figure. The predicted exposed (light blue), intermediate (white) and buried (yellow) residues exposed with more than 36%, 9-36%, and less than 9% of their surface, respectively. (**B**) The secondary structure composition of PGC7. (**C**) The percentage of exposed, intermediate and buried residues in PGC7.

No structural information is currently available for PGC7. To better understand its structural features, we predicted the secondary structure and solvent accessibility of mouse PGC7 using the PredictProtein server (https://www.predictprotein.org/) [47]. As shown in Figure 2A, PGC7 is predicted to have six helical regions (residues 5–7, 33–42, 73–81, 88–94, 97–104 and 107–111) and one β-strand (residues 127–132). The predictions for solvent accessibility for all residues are shown at the bottom of Figure 2A. Here, the predicted relative solvent accessibility was divided into 3 states: exposed, intermediate and buried, where residues exposed more than 36%, 9–36% and less than 9% of their surface, respectively. Figure 2B represents the secondary structure composition of mouse PGC7 and clearly shows that the loop structure (previously classified as the ‘random’ conformation) is the main secondary structure (68%). Helical structures (including the α-, π- and 3_10-helix) cover 28% of the protein length. Only six residues were predicted to form a β-strand. Figure 2C shows that more than half (59%) of the residues exposed more than 36% of their surface, and only 14% of the residues exposed less than 9% of their surface. This clearly indicates that the PGC7 protein tends to unfold and have a large exposed interface. 

### 2.2. Zinc Finger Protein 57 (ZFP57)

Zinc finger protein 57 (ZFP57) is another important maternal-effect protein, which plays a critical role in maintaining DNA methylation at multiple imprinted regions in mice and humans [48,49]. Deletion of maternal-zygotic ZFP57 in mouse embryos promotes loss of methylation at both paternally and maternally methylated imprinting control regions (ICRs), with no animals surviving to birth [48]. Mutations in the human ZFP57 coding sequence are also associated with aberrant hypomethylation at several ICRs, ultimately leading to disease [49,50,51,52,53].

**Figure 3:**
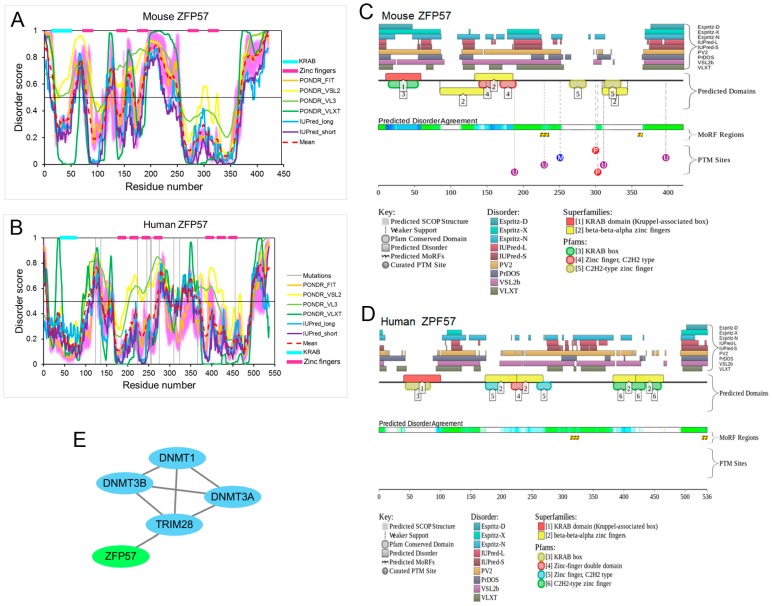
Functional disorder in mouse and human zinc finger protein 57 (ZFP57) proteins. (**A**,**B**) Disorder profiles generated by PONDR^®^FIT, PONDR^®^VSL2, PONDR^®^VL3, PONDR^®^VLXT, IUPred-long, and IUPred-short and a consensus disorder profile. Detailed statistical information is listed in supplemental Table S1. (**C**,**D**) Intrinsic disorder propensity and some important disorder-related functional information generated by the D^2^P^2^ database. **E.** The interaction network of ZFP57 with its partners. ZFP57 is known to physically interact with TRIM28. TRIM28 mediates the interaction between ZFP57 and three DNA methyltransferase (cytosine-5) 1 (DNMTs).

Mouse ZFP57 (Uniprot ID: Q8C6P8) is a 421 amino acid-long protein that contains one Kruppel-associated box (KRAB) domain and five zinc fingers (Figure 3A), whereas the human ZFP57 protein (Uniprot ID: B7ZW61) comprises 536 amino acids with one KRAB domain and seven zinc fingers (Figure 3B). Mouse and human ZFP57 proteins are highly conserved in their N-terminal KRAB boxes and the two middle zinc fingers [6,48,54,55]. To illustrate the intrinsic disorder propensity of ZFP57 protein, Figure 3A shows that mouse ZFP57 is predicted to have several IDPRs (residues 1–19, 54–79, 117–133, 153–168, 181–251 and 368–421), with the longest one being in the middle domain. The disorder distribution of human ZFP57 is moderately similar to mouse ZFP57, with four predicted long IDPRs (residues 104–139, 270–299, 329–370 and 495–536), and the longest two are located in the middle and C-terminal domains (Figure 3B). According to the average disorder scores, 48.45% of mouse ZFP57 and 30.41% of human ZFP57 residues are predicted to be disordered. Furthermore, the consensus MobiDB CPDR values of mouse and human ZFP57 are 45.37% and 25.75%, respectively. This suggests that the mouse ZFP57 protein is highly disordered (CPDR ≥ 30%), and the human ZFP57 protein is moderately disordered (10% ≤ CPDR < 30%).

The D^2^P^2^ outputs for mouse and human ZFP57 proteins are shown in Figure 3C,D, respectively. Apart from the intrinsic disorder propensity prediction, the D^2^P^2^ profile also shows two disorder-based protein binding sites (residues 223–235 and 358–364) and seven post-translational modifications (PTMs) in mouse ZFP57 (Figure 3C). In the D^2^P^2^ profile of human ZFP57, there are only two disorder-based protein binding sites (residues 312–326 and 527–536) (Figure 3D). No post-translational modification information is currently available for human ZFP57. In fact, there is also no correlative literature for the effect of various PTMs on the function of ZFP57. In agreement with the few disorder-based protein binding sites in ZFP57 proteins, Figure 3E shows that ZFP57 only interacts with one protein. In the study conducted by Zuo et al. (2012) [7], it was confirmed that the KRAB domain of ZFP57 is responsible for interaction with TRIM28 (also known as KAP1 or TIF1β), and TRIM28 mediates the interaction between ZFP57 and all three catalytically active DNMTs [6,7].

**Figure 4:**
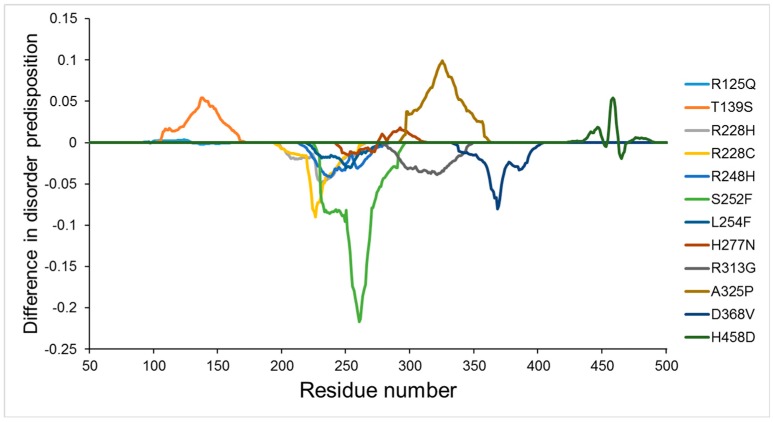
Effect of missense mutations on local intrinsic disorder propensity of human zinc finger protein 57 (ZFP57). The “difference disorder spectra” calculated as a simple difference between the per-residue disorder propensities of mutant and wildtype human ZFP57. The intrinsic disorder score of each residue were evaluated by the PONDR^®^VSL2.

Unlike the PGC7 protein, there are 12 human ZFP57 mutations that have been described in different human diseases, such as transient neonatal diabetes (TND) [49,50,51,52,53] and Silver-Russell syndrome [53]. As shown in Figure 3B, these mutations are mainly distributed in the middle domain, which contains several zinc fingers. To illustrate the effect of these mutations on disorder propensity of human ZFP57, Figure 4 shows the “disorder difference spectra” calculated as a simple difference between the disorder curves calculated for mutant and wildtype ZFP57. In this figure, the positive and negative peaks represent mutations causing a local increase or decrease, respectively, in intrinsic disorder propensity. It has been reported that two of these point mutations (R248H and H277N) affect ZFP57 binding to methylated DNA [50]. Correspondingly, Figure 4 shows that the R248H mutation decreased the local disorder propensity, and the H277N mutation caused opposite changes on both sides of the mutation site. In addition, there are also several point mutations that had significant effects on the local disorder propensity of human ZFP57, even though the effects of these mutations on the function of ZFP57 have not yet been identified [51,52].

**Figure 5:**
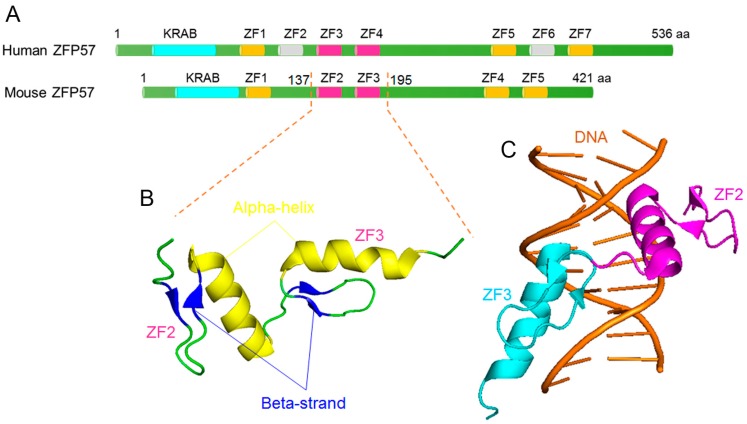
Structural characterization of zinc finger protein 57 (ZFP57) proteins. (**A**) Schematic diagrams are shown for human and mouse ZFP57 proteins. Same colours indicate the conserved domains between human and mouse ZFP57. Both human ZFP57 and mouse ZFP57 contain a highly conserved Kruppel-associated box (KRAB) (in cyan) at the N-terminus. The full-length human ZFP57 is predicted to have seven zinc finger (ZF) domains, whereas mouse ZFP57 only has five ZF domains. ZF3 and ZF4 of human ZFP57 are highly homologous to ZF2 and ZF3 of mouse ZFP57. (**B**) X-ray crystal structure of the DNA-binding domain of mouse ZFP57 (residues 137-195; PDB ID: 4GZN). Here, α-helices and β-strand are coloured in *yellow* and *blue*, respectively. (**C**) X-ray crystal structure of the DNA-binding domain of mouse ZFP57 (residues 137-195; PDB ID: 4GZN) in a complex with methylated DNA. The ZF2, ZF3 and methylated DNA are coloured in *magenta*, *cyan* and *orange*, respectively.

Figure 5 represents the structural information for ZFP57 proteins. The third (ZF3) and fourth (ZF4) zinc fingers of the human ZFP57 protein are highly conserved in mouse (corresponding to the second (ZF2) and third (ZF3) zinc fingers of mouse ZFP57 protein; Figure 5A) and are responsible for binding to a consensus hexanucleotide sequence ‘TGCCGC’ [6,48,50,54]. In fact, the structural information on ZFP57 protein is limited to the X-ray crystal structure of the DNA-binding domain of mouse ZFP57 (residues 137–195; PDB ID: 4GZN) in a complex with methylated DNA (Figure 5C) [54]. The two ZFs in mouse ZFP57 adopt the classical β-β-α motif (Figure 5B), positioning the α-helices for making canonical major groove interactions with three base pairs per zinc finger [54,56]. Mutation of the key residues in the two ZFs domain will alter the ZFP57-DNA binding capability [50,55,57]. It has been reported that two point mutations (R248H and H277N) affect ZFP57 binding to methylated DNA [50]. A single-base substitution mutation (E182Q) result in mouse ZFP57 gaining a significant binding affinity for 5-carboxylcytosine DNA [57].

### 2.3. Tripartite Motif-Containing 28 (TRIM28)

Tripartite motif-containing 28 (TRIM28), also known as KAP1 and TIF1β, is required for the maintenance of genomic imprinting [6,58,59]. TRIM28 is a maternal-effect protein, as indicated by the highly early embryonic lethality resulting from the dysregulation of genomic imprinting in mice lacking maternal TRIM28 [59,60]. It has also been reported that maternal TRIM28 not only maintains DNA methylation at germline imprints during early genome-wide reprogramming, but also regulates DNA methylation at imprinted gene promoters after genome-wide reprogramming [61].

Mouse TRIM28 is a multi-domain protein that is 834 amino acids long with a molecular mass of 89 kDa. It has a RING finger, two B-boxes and a coiled-coil region at the N-terminus, constituting the RBCC (RING finger, B boxes and coiled-coil) domain, which binds to KRAB proteins [62]. An HP1-binding motif (PXVXL) present in the middle of TRIM28 is responsible for binding to HP1 [63]. At the C-terminus are two adjacent domains: the plant homeodomain (PHD) finger and the bromodomain, which are essential for TRIM28 binding to the nucleosome remodeling and histone deacetylase (NuRD) complex and the histone methyltransferase, SETDB1(SET domain bifurcated 1) [64]. 

**Figure 6:**
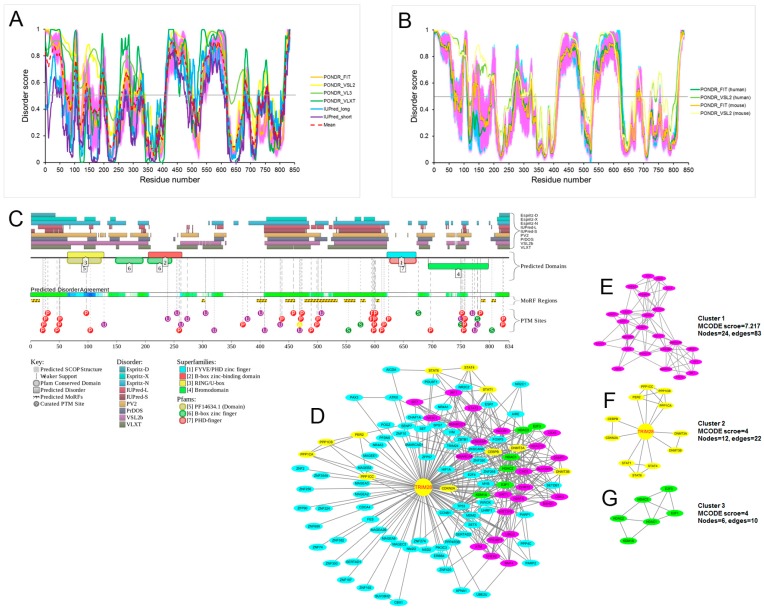
Functional disorder in mouse tripartite motif-containing 28 (TRIM28) protein (UniProt ID: Q62318). (**A**) Disorder profiles generated by PONDR^®^FIT, PONDR^®^VSL2, PONDR^®^VL3, PONDR^®^VLXT, IUPred-long, and IUPred-short and a consensus disorder profile. Detailed statistical information is listed in supplemental Table S1. (**B**) Analysis of the evolutionary conservation of intrinsic disorder propensity in TRIM28 proteins from *Mus musculus* (UniProt ID: Q62318) and *Homo sapiens* (UniProt ID: Q13263). (**C**) Intrinsic disorder propensity and some important disorder-related functional information generated by the D^2^P^2^ database. (**D**) The interaction network of TRIM28 with its partners. In this network, 113 previously validated TRIM28-interacting proteins were included. The STRING online tool was used to determine the known interactions between TRIM28 and its partners. The network was visualized in Cytoscape (version 3.4.0). (**E**–**G**) The three tightly connected network clusters obtained with MCODE are colour-coded and rendered as separate modules (MCODE scores ≥ 4 and nodes ≥ 6).

Figure 6A shows that mouse TRIM28 (UniProt ID: Q62318) is predicted to have four long IDPRs (residues 1–74, 260–289, 410–496 and 529–618), with the longest two being both sides of the HP1-binding domain. It is known that mouse and human TRIM28 proteins are highly conserved. Figure 6B shows the disorder profiles of mouse and human TRIM28 (UniProt ID: Q13263) and illustrates the remarkable similarity of these major order-disorder features. Furthermore, the CPDR values evaluated for these two proteins by the MobiDB platform were also similar (35.85% and 35.93% for mouse and human proteins, respectively). This clearly indicates that both mouse and human TRIM28 proteins are predicted to be highly disordered (CPDR > 30%).

According to the output of the D^2^P^2^ database (Figure 6C), mouse TRIM28 is predicted to have 10 MoRF regions (residues 1–15, 298–303, 394–411, 444–460, 477–534, 546–565, 575–583,602–607, 785–792 and 803–810), with most of them being concentrated within the two longest disordered regions. Further, this protein is extensively decorated with a multitude of different PTMs, such as phosphorylation, ubiquitination, SUMOylation and acetylation (Figure 6C). Numerous studies have shown that the phosphorylation of Tyr-449, Tyr-458, Ser-473 and Tyr-517, and the SUMOylation of Lys-779, Lys-804 and Ser-824 play critical roles in regulating the activity of TRIM28 [64,65,66,67,68,69,70]. However, there is no correlative literature reporting that PTMs affect the activity of TRIM28 on maintenance of DNA methylation. 

According to the MobiDB analysis, mouse TRIM28 interacts with 282 proteins, more than half of which were predicted to be highly or moderately disordered (170 partners were characterised by a CPDR > 10%), whereas MobiDB lists 607 proteins interacting with human TRIM28. There were 147, 183 and 277 TRIM28 partners that had a CPDR ≥ 30%, 10% ≤ CPDR < 30% and CPDR < 10%, respectively. To further elucidate the functional relationships between the TRIM28-interacting proteins and identify specific functional complexes, we mapped TRIM28 and its physically interacting interactors using the STRING interaction database. A protein–protein interaction (PPI) network containing 114 nodes and 391 edges was constructed (Figure 6D) with a medium confidence (confidence score ≥ 0.4) and limited active interaction sources (only experiments and databases were included). Moreover, we further analysed the interaction network for densely connected regions using the MCODE plugin tool in Cytoscape [71]. As shown in Figure 6E–G, three highly connected clusters were identified from the interaction network with MCODE scores ≥ 4 and nodes ≥ 6. Interestingly, all three interaction clusters we identified consisted of various effector proteins. This analysis aligned with previous studies: TRIM28 acts as a scaffold for chromatin-modifying complexes that comprise DNA methyltransferases [6,7], histone deacetylases [72], histone methyltransferases [73] and heterochromatin protein 1 [74]. It is probable that this complex is responsible for the maintenance of DNA methylation, although whether one or more of these effectors is recruited by TRIM28 is not yet known.

**Figure 7:**
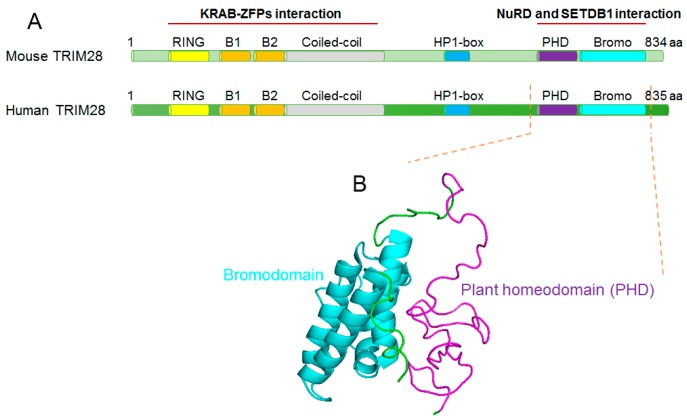
Structural characterization of tripartite motif-containing 28 (TRIM28) proteins. (**A**) Schematic diagrams are shown for mouse and human TRIM28 proteins. Conserved motifs include the RING finger, B boxes (B1 and B2), coiled-coil, HP1 binding box (HP1-box), plant homeo domain (PHD), and bromodomain (Bromo). The KRAB-ZFPs binding domain comprises the N terminal RBCC (RING finger, B boxes and coiled-coil) domain, while the NuRD and SETDB1 interaction domains are at the C terminus. (**B**) The nuclear magnetic resonance (NMR) solution structures of human TRIM28 PHD finger-bromodomain (residues 624–811; PDB ID: 2RO1). The plant homeodomain (PHD) and Bromodomain are coloured in *magenta* and *cyan*, respectively.

Figure 7 shows the structural information for TRIM28 proteins. Both the ordered and disordered regions are highly conserved in mouse and human TRIM28 proteins (Figure 7A). More detailed structural information is currently available for one fragment of human TRIM28. Zeng et al. [65] presented the nuclear magnetic resonance (NMR) solution structures of the human TRIM28 PHD finger-bromodomain (residues 624–811; PDB ID: 2RO1; Figure 7B). In this study, Zeng et al. also demonstrated that the PHD finger-bromodomain is required for TRIM28 co-repressor activity in gene silencing, through a comprehensive mutation-based structure-function analysis [65].

### 2.4. DNA Methyltransferase (Cytosine-5) 1 (DNMT1)

DNA methyltransferase (cytosine-5) 1 (DNMT1), is an important maternal-effect protein [8] and is the major enzyme responsible for the maintenance of DNA methylation in the genome. During each cellular DNA replication cycle, DNMT1 accurately restores the lineage-specific DNA methylation pattern on the daughter strand in accordance with that of the parental DNA [75]. Dysregulation of DNMT1 activity is involved in various diseases [76].

DNMT1 is a large molecule, comprising 1620 amino acid residues in mice and 1616 amino acid residues in humans. It has an N-terminal regulatory domain and a C-terminal catalytic domain linked by flexible lysine-glycine (KG) repeats. In the N-terminal domain, there are multiple regulatory motifs: DMAP1 and PCNA interaction domains [77,78], a replication foci-targeting sequence (RFTS) [79], a Zn-finger-like CXXC motif (CXXC) [80] and two bromo-adjacent homology (BAH) domains [81]. Studies have shown that DNMT1 activity is precisely regulated via physical interactions with diverse proteins and various PTMs [82]. Alterations in the N-terminal domain of DNMT1 have been implicated in dysregulation of genomic methylation in several studies [83,84,85].

**Figure 8:**
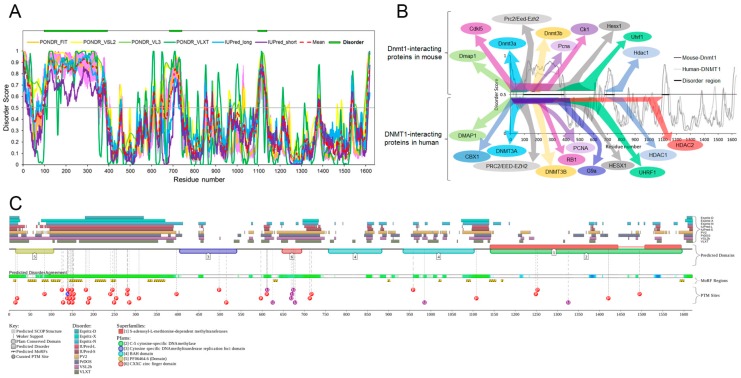
Functional disorder in mouse DNA methyltransferase (cytosine-5) 1 (DNMT1) protein (UniProt ID: P13864). (**A**) Disorder profiles generated by PONDR^®^FIT, PONDR^®^VSL2, PONDR^®^VL3, PONDR^®^VLXT, IUPred_long, and IUPred_short and a consensus disorder profile. Detailed statistical information is listed in supplemental Table S1. (**B**) Disorder-related interactivity analysis. A consensus disorder profile is shown in the centre of the figure along with 12 mouse proteins and 14 human proteins physically interact with the different disorder regions of DNMT1. (**C**) Intrinsic disorder propensity and some important disorder-related functional information generated the D^2^P^2^ database.

In the study conducted by Takeshita et al. [86], the crystal structure of the large fragment (residues 291–1620) of mouse DNMT1 was investigated in detail. The N-terminus of DNMT1 (residues 291–356) was identified as a disorder region. Here, our further multi-parametric computational analyses revealed that mouse DNMT1 is predicted to have several long IDPRs (residues 92–391, 678–740 and 1097–1139), and the longest one is located in the N-terminal domain (Figure 8A). The primary sequence of DNMT1 is highly conserved among various species. As shown in Figure 8B, mouse and human DNMT1 have almost the same long IDPR. Furthermore, the consensus MobiDB CPDR values of mouse DNMT1 (UniProt ID: P13864) and human DNMT1 (UniProt ID: P26358) are similar (30.12% and 30.26% for mouse and human proteins, respectively).

Figure 8B also shows 12 mouse and 14 human DNMT1 interacting proteins, all of which physically interact with the IDPRs of DNMT1 [77,78,82,87,88,89]. The abundance of protein–protein binding domains in the IDPRs of DNMT1 indicates that the disorder region is potentially involved in the regulation of DNMT1 function, a fact that has been confirmed by previous reports [90,91]. In addition, according to the MobiDB analysis, mouse DNMT1 interacts with 258 proteins; 53 were predicted to be highly disordered (CPDR ≥ 30%), 53 moderately disordered (10% ≤ CPDR < 30%) and 152 highly ordered (CPDR < 10%). Human DNMT1 interacts with 387 proteins; 106 were predicted to be highly disordered (CPDR ≥ 30%), 71 moderately disordered (10% ≤ CPDR < 30%) and 210 highly ordered (CPDR < 10%).

To further understand the functionality of intrinsic disorder in DNMT1, Figure 8C shows that mouse DNMT1 is predicted to have 16 MoRFs ranging in length from 6 to 32 residues (residues 10–16, 44–64, 77–93, 99–110, 142–173, 203–229, 241–252, 328–339, 343–369, 626–636, 897–903, 1018–1024, 1084–1094, 1138–1154, 1166–1171 and 1590–1600). Importantly, half of the MoRFs are concentrated within the longest IDPR. It implies that this IDPR is a platform for interaction with many factors and in fact, there are many different proteins binding to this region [77,78,82,87,88,89].

DNMT1 protein has numerous PTMs, including phosphorylation, methylation, ubiquitination and acetylation (Figure 8C); however, only a few of them have been functionally studied. Phosphorylation of mouse DNMT1 at Ser-146 by CK1 (casein kinase 1) decreases the DNA-binding affinity [92], phosphorylation of mouse DNMT1 Ser-152 (or human DNMT1 Ser-154) by cyclin-dependent kinases increases the enzymatic activity [93,94] and protein stability and phosphorylation of human DNMT1 at Ser-127 and Ser-143 by AKT1 (v-akt murine thymoma viral oncogene homolog 1) decreases the interaction of DNMT1 with PCNA and UHRF1 [95]. Further, methylation of Lys-142 on human DNMT1 by SET domain containing lysine methyltransferase 7 (SETD7) leads to DNMT1 degradation [96]. Phosphorylation of Ser-143 by AKT1 prevents methylation by SETD7, thereby increasing DNMT1 stability [97]. Interestingly, all of the above-mentioned post-translational modification sites are located in the IDPR of DNMT1, indicating that the conserved and abundant disorder plays an important role in regulating the functions of DNMT1.

In addition to PTMs, mutations in the IDPRs also significantly affect the function of DNMT1 in maintaining DNA methylation. Borowczyk et al. (2009) [84] found that different deletion mutations in the disorder region of DNMT1 selectively regulated the maintenance of different types of genomic methylation patterns. Four DNMT1 mutants (Δ222–258, Δ255–291, Δ288–300 and Δ305–317) were unable to maintain differentially methylated domains (DMDs) and non-DMD methylation. The DNMT1 mutant (Δ297–309) maintained DMD methylation but not non-DMD methylation. In contrast, the Δ191–324 mutant maintained non-DMD methylation but not DMD methylation. In the study conducted by D’Aiuto et al. (2010) [83], regional frame-shift mutagenesis (RFM) among amino acids 124–160 (RFM4), 386–404 (RFM12A) and 698–740 (RFM23) abolished the methyltransferase activity of DNMT1, whereas RFM7 (241–276), RFM8 (276–303) and RFM9 (303–340) mutant proteins could maintain genomic methylation. Shaffer et al. (2015) [85] also reported that two mouse lines (Dnmt1^IDel13/IDel13^ and Dnmt1^P/P^) with DNMT1 IDPR mutations had alterations in imprinted and/or non-imprinted (global) DNA methylation. 

**Figure 9:**
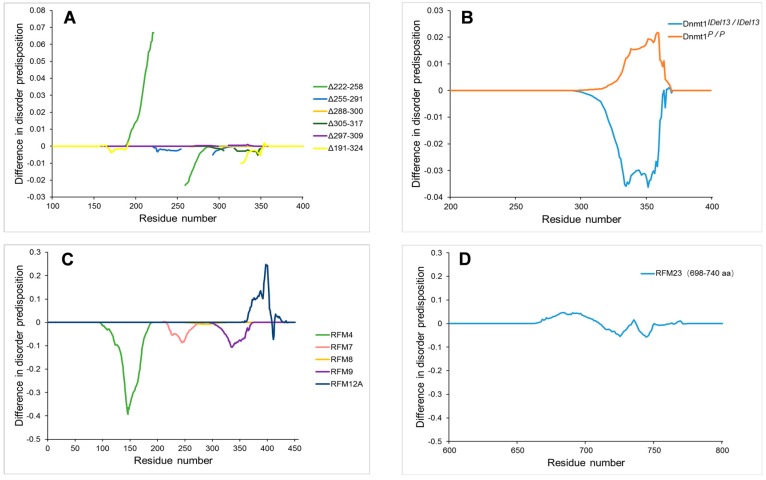
Difference disorder spectra calculated based on the effect of various mutations on intrinsic disorder propensity of mouse DNA methyltransferase (cytosine-5) 1 (DNMT1). The effects of six deletion mutations (**A**) two substitution mutations (**B**) and six regional frame-shift mutagenesis (RFM) mutations (**C**,**D**) on the local disorder propensity of mouse DNMT1.

Figure 9 shows the effect of the above-mentioned mutations on the local disorder propensity of mouse DNMT1 in the form of “disorder difference spectra”. Among the six deletion mutations, only the Δ222–258 mutant showed a noticeable change in intrinsic disorder propensity (Figure 9A). In Shaffer’s study [85], the amino acid sequence ‘RRKTTRKKLESHTV’ (319–333) was replaced by “DEKRHVKNWSHTPF” in the Dnmt1I^Del13/IDel13^ mutant mouse line and the amino acid sequence “LESHTV” (328–333) was replaced by “PEPLSI” in the Dnmt1^P/P^ mutant mouse line. As shown in Figure 9B, the two mutations differently affected the local disorder score. The Dnmt1^IDel13/IDel13^ mutation caused a decrease in local disorder propensity, whereas the Dnmt1P/P mutation was associated with a local increase in the intrinsic disorder propensity. Here, we also analysed the effects of RFM mutations on the disorder predisposition of mouse DNMT1.

Interestingly, RFM4 and RFM12A mutations, which result in loss of the methyltransferase ability of DNMT1, caused significant changes in the local disorder propensity. The RFM7, RFM8 and RFM9 mutations, which do not abolish the enzyme activity of DNMT1, were associated with a relatively slight decrease in the intrinsic disorder propensity (Figure 9C). In regard to the RFM23 mutation, the mutant DNMT1 protein was unable to maintain genomic methylation even though this mutation induced less change in the local disorder propensity (Figure 9D). To investigate the reason, we examined the location of the RFM23 mutation and found that it is located in the disordered linker between the CXXC and BAH1 domains.

**Figure 10:**
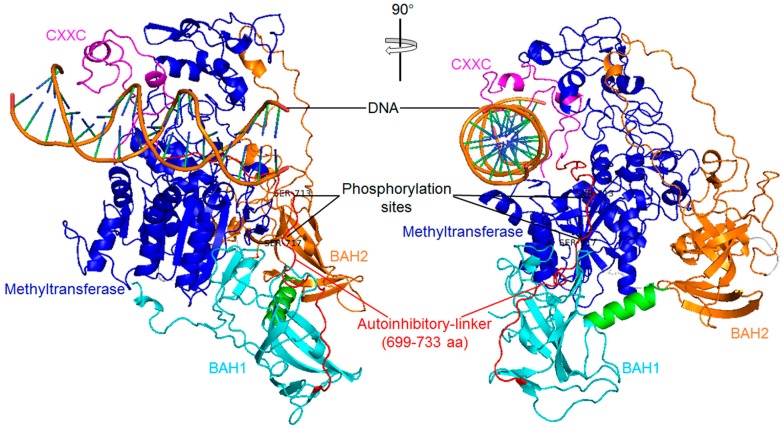
Structural characterization of mouse DNA methyltransferase (cytosine-5) 1 (650–1602) in complex with DNA (PDB ID: 3PT6). Ribbon representation of the complex in two orthogonal views. The zinc finger-like motif (CXXC), bromo-associated homology domain 1 (BAH1) and 2 (BAH2), and methyltransferase domain are coloured in *magenta*, *cyan*, *orange* and *blue*, respectively. The autoinhibitory linker (CXXC-BAH1 linker) is coloured in *red*. Two phosphorylation sites (SER-713 and SER-717) are also shown in the figure.

Several X-ray crystal structures of the DNMT1 fragment are currently available. Figure 10 shows the crystal structure of mouse DNMT1 (650–1602) in complex with DNA (PDB ID: 3PT6). Here, we observed that the CXXC domain and CXXC-BAH1 domain linker (an autoinhibitory linker) occluded DNA from the methyltransferase domain (Figure 10). In 2011, Song et al. [98] reported that the autoinhibitory linker plays a key role in inhibiting the enzymatic activity of mouse DNMT11 through a multi-layered mechanism. Later, Zhang et al. (2015) demonstrated that the role of the autoinhibitory linker in human DNMT1 is similar to that of mouse DNMT1, while a deletion mutation of the autoinhibitory linker (Δ694–701) changes the conformation of human DNMT1 and increases its DNA methylation activity towards 12 base pair DNA [99]. In addition, we found that there are two predicted phosphorylation sites (Ser-713 and Ser-717) located within the autoinhibitory linker region (Figure 10). Although there have not been any related studies, we suspect that the two phosphorylation sites may play a role in regulating the activity of DNMT1 because the two phosphorylation sites are mutated in the regional frame-shift mutagenesis (RFM) 23 mutant DNMT1 protein [83], which is unable to maintain DNA methylation [83].

## 3. Materials and Methods

Amino acid sequences and some structure/function information for all proteins analysed in this study were retrieved from UniProt or NCBI databases. The intrinsic disorder propensities of eight proteins, including mouse PGC7 (UniProt ID: Q8QZY3), human PGC7 (UniProt ID: Q6W0C5), mouse ZFP57 (Uniprot ID: Q8C6P8), human ZFP57 protein (Uniprot ID: B7ZW61), mouse TRIM28 (UniProt ID: Q62318), human TRIM28 (UniProt ID: Q13263), mouse DNMT1 protein (UniProt ID: P13864) and human DNMT1 (UniProt ID: P26358), were analysed in this study.

A general characteristic of IDPs is the presence of distinct amino acid compositional biases and low sequence complexity [16,23,36]. They have a higher proportion of disorder-promoting amino acids (A, E, G, K, P, Q, R and S), and a low content of order-promoting amino acids (C, F, I, L, N, V, W and Y) [15,23,100]. According to this description, we analysed the amino acid composition of PGC7 from 10 representative species. 

CH (charge–hydrophobicity) plots were generated as described by Uversky et al. (2002) [101]. Intrinsically disordered proteins were separated from ordered proteins by a linear boundary, with disordered proteins above the boundary and ordered proteins below. This boundary is described by the relation: Hb = (R + 1.151)/2.785, where Hb stands for the ‘boundary’ mean hydrophobicity value and R for mean net charge [101]. The mean hydrophobicity and mean net charge values of each amino acid sequence were calculated with PONDR (http://www.pondr.com/).

According to the method described by Uversky et al. (2017) [102,103], the intrinsic disorder propensities of target proteins were evaluated using four algorithms from the PONDR family (PONDR-FIT, PONDR^®^VSL2, PONDR^®^VL3 and PONDR^®^VLXT) [36,37,38,39,40], as well as IUPred-long and IUPred-short [41]. The mean intrinsic disorder value of each protein was calculated by averaging the disorder profiles of the above six predictors. We further retrieved disorder information for target proteins using the MobiDB database (http://mobidb.bio.unipd.it/) [42,43], which aggregates the outputs from 10 disorder predictors, such as DisEMBL in two of its flavours, ESpritz in its three flavours, GlobPlot, IUPred in its two flavours, JRONN and VSL2b. MobiDB also has additional annotations related to protein structure and interaction derived from UniProt [104], Pfam [105], PDB [106] and STRING [107]. In addition, D^2^P^2^ is a database of disordered protein predictions (http://d2p2.pro/) [45]. This database uses the outputs of ESpritz [108], IUPred [41], PV2 [45], PrDOS [109], PONDR VSL2b [39,40] and PONDR VLXT [36] to evaluate the intrinsic disorder propensity of target proteins. Some important disorder-related functional information, including various post-translational modifications and predicted disorder-based protein binding sites, were also retrieved from the D2P2 database [45].

The STRING database (version 10.5) [107] is an integrated protein interaction database that has been designed to evaluate known and predicted protein–protein interaction (PPI) information. In this study, the maternal-effect proteins and their binding partners were searched against the STRING database for protein–protein interactions. STRING uses a metric called ‘confidence score’ to define interaction confidence; we chose a confidence score ≥ 0.4 (medium confidence) as the cut-off criterion. There are seven types of evidence that are used to build the corresponding network. However, only experimental and database evidence were selected in our analysis. The maximum number of interactors selected in both the first and second shells of the network were zero. All of the interaction networks were generated in this manner and then visualised by Cytoscape v3.4.0. The interaction network was further analysed for highly connected clusters using the program “Molecular Complex Detection” (MCODE) [71]. Default parameters (Degree Cut-off: 2, Node Score Cutoff: 0.2, K-Core: 2, Max. Depth from seed: 100) were used as the cut-off criteria for network module screening.

We characterised the secondary structure and solvent accessibility of mouse PGC7 using the PredictProtein server (https://www.predictprotein.org/) with default parameters [47]. The structural characterization of ZFP57, TRIM28 and DNMT1 were retrieved from the PDB and represented by using the Education-Use-Only PyMOL tool (http://www.pymol.org/).

## 4. Conclusions

DNA methylation is an important epigenetic modification that plays a critical role in normal mammalian development and disease. Maternal-effect proteins such as PGC7, ZFP57, TRIM28 and DNMT1 are now associated with the maintenance of DNA methylation during the development of preimplantation embryos. PGC7 has been shown to protect DNA methylation from Tet-mediated 5-mC to 5-hmC conversion through suppressing the enzymatic activity of TET2 and TET3 [4,5], whereas the other three proteins, ZFP57, TRIM28 and DNMT1 appear to play key roles in protecting against passive demethylation during preimplantation development. However, the molecular mechanisms by which these maternal-effect proteins maintain DNA methylation are not yet fully understood.

Here, we started from a new perspective to understand these maternal-effect proteins and discuss the effect of their intrinsic disorder on the maintenance of DNA methylation. Based on the comprehensive intrinsic disorder analysis and the intensive literature search for structure information, we found that the disorder regions potentially regulate the functions of the four maternal-effect proteins. In fact, the IDPRs of DNMT1 (residues 92–391) participate in the regulation of its DNA methyltransferase activity, which has been confirmed by previous reports [90,91]. In addition, various mutations and PTMs at the intrinsic disorder regions of these maternal-effect proteins may directly affect their functions. For example, it has been described that a point mutation (H277N) affects human ZFP57 binding to methylated DNA [50], while phosphorylation in the IDPRs of DNMT1 significantly affect its function in maintaining DNA methylation [83,85,93,94].

One way to understand the function of a protein is to identify its interacting partners. In this article, we also conducted an intensive literature search to gain information on the interaction of the four maternal-effect proteins. As PGC7, ZFP57 and TRIM28 are not equipped with known enzymatic domains, it is their interacting partners that assist in regulating DNA methylation. A number of proteins that interact with the IDPRs of DNMT1 have also been described as controlling the enzymatic activity of DNMT1. 

According to the literature, there are only four proteins (IPO5, UHRF1, TET2 and TET3) that interact with PGC7 [4,5,33]. This severely limits our ability to understand the molecular mechanisms by which PGC7 regulates DNA methylation. Fortunately, in our proteome research, 290 proteins were identified that interact with PGC7 [46]. Figure 1E shows 23 newly validated PGC7-interacting proteins [46]. Among the 23 interacting partners, DMAP1 is most likely the one involved in the regulation of DNA methylation because we found that DMAP1 mediates the interaction between PGC7 and DNMT1. Moreover, our proteomic analysis also showed that PGC7 is linked to the cell cycle. All of this data prompts us to speculate that PGC7 may play a role in regulating DNA methylation during cell division. To some degree, the fact that PGC7 overexpression in NIH3T3 cells induced global DNA demethylation supports this hypothesis [33].

**Figure 11:**
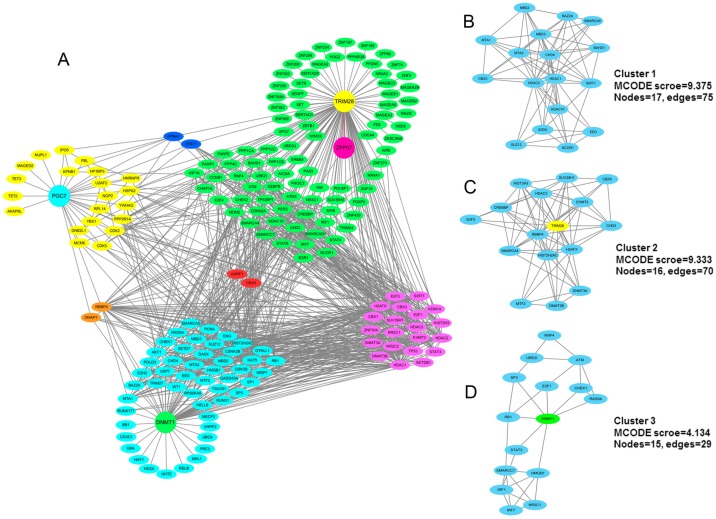
The interaction network of four maternal-effect proteins and their partners. (**A**) In this network, four maternal-effect proteins (PGC7, ZFP57, TRIM28 and DNMT1) and their physical interacting proteins were included. The STRING online tool was used to determine the known interactions between these proteins. A protein–protein interaction (PPI) network containing 189 nodes and 927 edges were constructed, and then further visualized in Cytoscape (version 3.4.0). The two proteins interact with both PGC7 and TRIM28 are coloured in *blue*; two proteins interact with both PGC7 and DNMT1 are coloured in *orange*; 21 proteins interact with both TRIM28 and DNMT1 are coloured in *purple*; and two proteins interact with three of the maternal-effect proteins (PGC7, TRIM28 and DNMT1) are coloured in *red*. The interacting partners of four maternal-effect proteins are list Supplementary Table S2. (**B**–**D**) The three tightly connected network clusters obtained with MCODE and rendered as separate modules (MCODE scores ≥ 4 and nodes ≥ 6).

To further illustrate the cross-interactivity of these four maternal-effect proteins and their binding partners, all of these proteins (including 27 PGC7, 113 TRIM28 and 72 DNMT1 interacting proteins) were subjected to a PPI network analysis using the STRING database. A PPI network containing 189 nodes and 927 edges was constructed (Figure 11A) with a medium confidence (confidence score ≥ 0.4) and limited active interaction sources (only experiments and databases were included). The resulting interactome clearly showed that most of the interacting partners for each maternal-effect protein communicated with each other. The three maternal-effect proteins PGC7, TRIM28 and DNMT1 also had two of the same interacting partners (UHRF1 and CBX5). According to the MCODE analysis, three highly connected clusters were identified from the PPI network (Figure 11B–D) and TRIM28 and DNMT1 were included in the tightly connected clusters 2 and 3, respectively.

Since three of the four maternal-effect proteins (except for ZFP57) analysed in this study directly bind to various partners and the three proteins are predicted to be highly disordered (CPDR > 30%), PGC7, TRIM28 and DNMT1 can be considered as disordered hub proteins. It is known that disordered hubs have the ability to adapt a variety of binding partners to perform different functions [11,21,110]. Many interacting partners were highly connected with the hub and formed a complex PPI network, which not only provides a valuable resource for understanding the diverse functions of this hub, but may also present a major challenge for the identification of targeting partners.

## Figures and Tables

**Table 1:** Analysis of disorder- and order-promoting residues in primordial germ cell 7 (PGC7).

PGC7 (Species)	Disorder-Promoting Residues (%)	Order-Promoting Residues (%)
Mouse mouse	57.33	28.67
Human	57.23	30.82
Norway rat	56.96	28.48
Cattle	52.15	31.28
Chimpanzee	57.23	30.82
Rhesus monkey	52.76	32.51
Horse	58.06	30.10
Rabbit	57.43	27.70
Pig	51.33	33.33
Goat	52.76	30.67

## Supplementary Materials

Supplementary materials can be found at www.mdpi.com/1422-0067/18/9/1898/s1.
